# Exploring KRAS-mutant pancreatic ductal adenocarcinoma: a model validation study

**DOI:** 10.3389/fimmu.2023.1203459

**Published:** 2024-01-10

**Authors:** Fan Yang, Yanjie He, Nan Ge, Jintao Guo, Fei Yang, Siyu Sun

**Affiliations:** ^1^ Department of Gastroenterology, Shengjing Hospital of China Medical University, Shenyang, China; ^2^ Department of Surgery, New York University School of Medicine and NYU-Langone Medical Center, New York, NY, United States

**Keywords:** pancreatic ductal adenocarcinoma, KRAS mutations, risk prediction model, ferroptosis, immune microenvironment, potential drugs screening

## Abstract

**Introduction:**

Pancreatic ductal adenocarcinoma (PDAC) has the highest mortality rate among all solid tumors. Tumorigenesis is promoted by the oncogene KRAS, and KRAS mutations are prevalent in patients with PDAC. Therefore, a comprehensive understanding of the interactions between KRAS mutations and PDAC may expediate the development of therapeutic strategies for reversing the progression of malignant tumors. Our study aims at establishing and validating a prediction model of KRAS mutations in patients with PDAC based on survival analysis and mRNA expression.

**Methods:**

A total of 184 and 412 patients with PDAC from The Cancer Genome Atlas (TCGA) database and the International Cancer Genome Consortium (ICGC), respectively, were included in the study.

**Results:**

After tumor mutation profile and copy number variation (CNV) analyses, we established and validated a prediction model of KRAS mutations, based on survival analysis and mRNA expression, that contained seven genes: CSTF2, FAF2, KIF20B, AKR1A1, APOM, KRT6C, and CD70. We confirmed that the model has a good predictive ability for the prognosis of overall survival (OS) in patients with KRAS-mutated PDAC. Then, we analyzed differential biological pathways, especially the ferroptosis pathway, through principal component analysis, pathway enrichment analysis, Gene Ontology (GO) enrichment analysis, and gene set enrichment analysis (GSEA), with which patients were classified into low- or high-risk groups. Pathway enrichment results revealed enrichment in the cytokine-cytokine receptor interaction, metabolism of xenobiotics by cytochrome P450, and viral protein interaction with cytokine and cytokine receptor pathways. Most of the enriched pathways are metabolic pathways predominantly enriched by downregulated genes, suggesting numerous downregulated metabolic pathways in the high-risk group. Subsequent tumor immune infiltration analysis indicated that neutrophil infiltration, resting CD4 memory T cells, and resting natural killer (NK) cells correlated with the risk score. After verifying that the seven gene expression levels in different KRAS-mutated pancreatic cancer cell lines were similar to that in the model, we screened potential drugs related to the risk score.

**Discussion:**

This study established, analyzed, and validated a model for predicting the prognosis of PDAC based on risk stratification according to KRAS mutations, and identified differential pathways and highly effective drugs.

## 1 Introduction

Pancreatic cancer is a highly malignant gastrointestinal cancer that can be difficult to diagnose and treat. Among the various subtypes of pancreatic cancer, pancreatic ductal adenocarcinoma (PDAC) represents 90% of pancreatic malignancies (1–3). In recent years, the morbidity and mortality associated with pancreatic cancer have increased considerably, with PDAC exhibiting the highest mortality rate among all solid tumors (3). Moreover, pancreatic cancer has a relatively low rate of early diagnosis and is often diagnosed at advanced stages, by which time the cancer has spread, becoming more difficult to treat and resulting in a 5-year survival rate of less than 7% (2, 4). PDAC is one of the malignant tumors with the worst prognosis. According to the latest United States Cancer Statistics, pancreatic cancer has the tenth and eighth highest incidence rate for males and females, respectively, as well as the fourth highest mortality rate for both males and females (2). Furthermore, the latest World Health Organization (WHO) statistics reveal that pancreatic cancer was the seventh most common cancer, and sixth most fatal cancer, in China in 2020 (1). Although there are several new treatment methods or targets in recent years, the prognosis of pancreatic ductal adenocarcinoma remains poor (1–5).

Distant metastasis occurs in approximately 50% of patients with PDAC (6). PDAC often metastasizes at early stages because of complex interactions between cell-autonomous processes and cellular components in the tumor microenvironment. The oncogene KRAS promotes tumorigenesis, whereas inactivation of key tumor suppressor genes accelerates the malignant progression of pancreatic intraepithelial neoplasia precursor lesions (7). KRAS mutations are present in approximately 90% of patients with PDAC (8). Hence, an in-depth understanding of the complex interactions between KRAS mutations and PDAC may facilitate the development of therapeutic strategies for reversing the progression of malignant tumors.

Nevertheless, KRAS has long been considered an undruggable target for the following reasons: (1) mutant KRAS is a GTPase with a high picomolar binding affinity with GTP; (2) the KRAS substrate, GTP, has a very high intracellular concentration; and (3) the KRAS protein has a very smooth surface that rarely harbors small-molecule binding pockets other than the GTP-binding site. These characteristics hinder the development of small-molecule inhibitors and prevent the direct, specific targeting of KRAS (8). Therefore, the research focus has shifted to indirect targets in cascades or pathways upstream and downstream of KRAS (e.g., blocking the RAF-MEK-MAPK pathway with MEK inhibitors), which is a promising approach (9). Several relevant pathway-targeting drugs have received approval from the Food and Drug Administration (FDA); however, their antitumor activities are limited by associated toxicity (10). As for direct KRAS inhibitors, significant progress has been made in their development and advancement to phase 1 clinical trials. Notably, sotorasib and adagrasib, two prominent direct KRAS inhibitors, have successfully moved into phase 1 clinical trials and have received FDA approval (10). These advancements emphasize the promising potential of direct KRAS inhibitors as effective treatment strategies for KRAS-related diseases. Consequently, a comprehensive analysis of additional KRAS-associated pathways, including the emerging role of ferroptosis, is crucial for establishing optimal therapeutic approaches that specifically target KRAS or its downstream effectors in tumors. This analysis is of significant importance for preserving the integrity of KRAS signal transduction in non-malignant tissues.

Ferroptosis, a recently identified iron-dependent form of regulated cell death, has emerged as a critical pathway in cancer biology and therapy. Dysregulation of ferroptosis has been implicated in various malignancies, including PDAC (11). Investigating the changes in ferroptosis-associated pathways is therefore essential for understanding the molecular mechanisms underlying KRAS-driven tumorigenesis and identifying effective therapeutic strategies. Furthermore, the interplay between KRAS-associated pathways, including ferroptosis, and the tumor microenvironment, particularly the immune response, is of great interest. By analyzing the immunocorrelation between these pathways and the tumor microenvironment, it is possible to gain valuable insights into the complex interactions between cancer cells and immune cells. This analysis can guide the identification of potential immunotherapeutic targets and facilitate the development of combinatorial strategies that leverage the antitumor immune response while targeting KRAS-driven pathways. Hence, considering the emerging significance of ferroptosis in cancer and its potential interplay with KRAS signaling, an in-depth analysis of changes in ferroptosis-associated pathways is warranted. This approach will enable the identification of novel therapeutic targets and the development of more effective treatments for PDAC, while ensuring the preservation of normal tissue homeostasis mediated by KRAS-related signaling.

Therefore, in-depth analysis of the differentially expressed genes (DEGs) related to KRAS mutations and changes in ferroptosis-associated pathways in patients with PDAC is required to facilitate the development of PDAC treatment strategies. In this study, we first subjected *PDAC* gene expression profiles and mutation data retrieved from public databases [i.e., The Cancer Genome Atlas (TCGA) and International Cancer Genome Consortium (ICGC)] to tumor mutation profile and copy number variation (CNV) analyses to establish a prediction model of KRAS mutations based on survival analysis and mRNA expression. After evaluating and validating the model, we used it to elucidate the KRAS mutation-related ferroptosis pathways via differential gene expression and pathway enrichment analyses. Finally, we analyzed the correlation between PDAC risk scores and the immune microenvironment and identify drug candidates for effective treatment against PDAC.

## 2 Materials and methods

### 2.1 Data retrieval and preprocessing

Pancreatic cancer-associated expression profiles and mutation datasets with a reliable source of samples (i.e., TCGA-PAAD) were downloaded from TCGA database using the R package TCGAbiolinks (version 4.0.2, http://r-project.org/). Additional expression profiles and mutation datasets of pancreatic cancer (i.e., PACA-AU) were retrieved from the ICGC database (https://dcc.icgc.org). All datasets were generated using samples derived from *Homo sapiens*. The expression profiles in the PACA-AU dataset were created using microarray data generated on the Illumina microarray platform GPL10558. TCGA-PAAD dataset comprises mutation and expression profiles for tumor samples from 184 patients with pancreatic cancer, whereas the PDAC-AU dataset contains mutation profiles for tumor samples from 412 patients, as well as gene expression profiles for tumor samples from 271 patients. Neoantigen counts of the TCGA-PAAD dataset were retrieved from The Cancer Immunome Atlas (https://tcia.at/home), whereas other indicators, such as the tumor mutation burden (TMB) and microsatellite instability (MSI), were retrieved from the cBioPortal website (http://www.cbioportal.org/). Tumors were staged according to the 8th tumor-node-metastasis (TNM) staging system drafted by the International Union Against Cancer. Identification of neoplasm histologic grades was performed using the World Health Organization (WHO) grading system for PDAC (12).

### 2.2 Tumor mutation profile and CNV analyses

Somatic mutation data retrieved from the public databases TCGA and ICGC were analyzed for the aforementioned 184 and 412 patients, respectively. After creating Mutation Annotation Format files of the somatic mutations, visualization was performed using the maftool package with multiple analysis modules (13) to display the somatic landscape. We then used the Genomic Identification of Significant Targets in Cancer (GISTIC) algorithm to detect common CNV regions shared across all samples with a q value < 0.05 in the TCGA-OV dataset, including chromosome arm-level CNVs and minimal common regions between samples (14).

### 2.3 Construction of the prediction model

ElasticNet is a regularization method extended from Ridge Regression and LASSO. The R package glmnet can be used to fit a generalized linear model with ElasticNet regularization (15). ICGC and TCGA data were employed as training and validation datasets, respectively, to evaluate the predictive ability of gene expression for KRAS mutations. To use the ElasticNet method, the expression data were filtered to include only genes shared between the two datasets, as these two datasets may contain expression values of slightly different gene sets. We used α = 0.9 as the ElasticNet penalty parameter to fit the generalized linear model and performed cross-validation using ElasticNet methods. Higher alpha values (α = 0.9) reduce the risk of overfitting, which is crucial when dealing with biological data where the number of features (genes) often exceeds the number of samples. It helps prevent the model from fitting noise in the data. One of the cross-validation scores is the regularization parameter (lambda), which determines the amount of shrinkage used to train the machine learning model. The machine learning classifier was trained using the training dataset via leave-one-out cross-validation then validated using the testing dataset (16).

### 2.4 Selection of prognostic markers

The correlation between the expression of each candidate gene and the overall survival (OS) was determined using the univariate Cox proportional-hazards regression model, with the ICGC dataset of samples with KRAS mutations being used as the training dataset. We used the LASSO algorithm for variable selection in a Cox regression model to identify significant prognostic genes, in accordance with the one-standard-error rule. Thereafter, we further optimized and improved the practicality of our model via stepwise Cox proportional-hazards regression analysis. This was achieved through feature selection, improving model parsimony, enhancing predictive capability, and determining the optimal subset. Finally, the risk score was calculated on the basis of the gene expression and estimated Cox regression coefficient as follows: Risk Score = (exp Gene1 × coef Gene1) + (exp Gene2 × coef Gene2) +…… + (exp Gene × coef Gene). The patients were then divided into a high-risk group and a low-risk group according to their risk scores. The OS rate of patients in each testing dataset was assessed via Kaplan–Meier analysis and a log-rank test using the ‘survival’ R package. Additionally, we evaluated the survival prediction using the time-dependent receiver operating characteristic (ROC) curves. The prognostic or predictive accuracy of markers was determined by calculating the area under the ROC curve (AUC) using the pROCR package (17), and validated using an independent TCGA dataset of KRAS mutations.

### 2.5 Correlation between risk factors and clinical parameters

According to clinical parameters, such as age and cancer stage, the risk score and other clinical parameters in the datasets were integrated using univariate and multivariate Cox regression analyses to assess the risk score as an independent prognostic predictor. A nomogram of statistically independent predictors was plotted using the rms package, and its predictive performance was assessed using calibration curves.

### 2.6 Screening and functional analysis of DEGs

The ICGC cohort of patients with KRAS mutations was stratified according to their risk coefficients to identify DEGs using the limma package. Volcano plots and a DEG heatmap were also plotted using the ‘ggplot2’ and ‘pheatmap’ R packages, respectively, to depict the differential expression of genes across the samples. The DEGs screened from the dataset and stratified according to the risk score met the criteria of differential expression, i.e., adjusted p value (p adj) < 0.05 and |log2FC| > 0.05. Subsequently, the associated genes were screened according to their correlation with the risk score. Similarly, volcano plots and a heatmap of associated genes were plotted using the ggplot2 package and pheatmap package, respectively, to illustrate the differential expression of the associated genes across the samples. The DEGs were then subjected to Gene Ontology (GO) and Kyoto Encyclopedia of Genes and Genomes (KEGG) pathway enrichment analyses using the ‘clusterProfiler’ package (18). The ‘clusterProfiler’ package was also used for gene set enrichment analysis (GSEA) of the gene expression matrix, using “c2.cp.kegg.v7.0.symbols.gmt” and “h.all.v7.2.symbols.gmt” as the reference gene sets. A false discovery rate (FDR) < 0.25 and *p* < 0.05 were considered to indicate a significantly enriched gene set. Subsequently, gene set variation analysis (GSVA) of the ICGC cohort of samples with KRAS mutations was performed using the R package ‘GSVA’ (19) to calculate the enrichment scores of each pathway based on the gene expression matrix of each sample. Statistical analysis was performed to determine the significance of differences in the enrichment score between different risk groups.

### 2.7 Immunocorrelation analysis and correlation with risk scores

CIBERSORT implements linear support vector regression to deconvolute the gene expression matrices generated from transcriptome data and estimate the composition and abundance of immune cells in a heterogeneous mixture of cells (20). In the present study, we uploaded the gene expression matrices to CIBERSORT, only including samples with *p* values < 0.05, to obtain the profiles of immune cell infiltration. Histograms depicting the distribution of 22 types of infiltrating immune cells across samples were plotted using the ‘ggplot2’ R package. Heatmaps illustrating the correlations between the risk score and the 22 types of infiltrating immune cells and the expression of human leukocyte antigen (HLA) family genes were plotted using the ‘corrplot’ package. The stromal score, immune score, ESTIMATE score, and tumor purity were calculated on the basis of the mRNA expression using the R package ESTIMATE. The correlation between the risk score and the immune-related gene datasets retrieved from the immortal database was analyzed and visualized using Cytoscape software (21).

### 2.8 qRT-PCR detection of mRNA expression related to prognosis model in pancreatic cancer cell lines with different mutation sites

Four types of KRAS-mutant human pancreatic cancer cell lines: MIA Paca-2(KRAS G12C mut), PANC-1(KRAS G12D mut), SW1990(KRAS G12D mut), Capan-2(KRAS G12V mut), and 1 type of KRAS wild-type (WT) human pancreatic cancer cell line BxPC-3 were purchased from the Shanghai Institutes for Biological Science Cell Resource Center. Total RNA from all cell lines was extracted using TRIzol reagent (Invitrogen, Carlsbad, CA, United States). Reverse transcription and real-time quantitative reverse transcription polymerase chain reactions (qRT-PCR) were performed to detect the mRNA expression levels of *CSTF2*, *FAF2*, *KIF20B*, *AKR1A1*, *APOM*, *KRT6C*, and *CD70*. For details, see **Supplementary File 1**.

### 2.9 Prediction of drug efficacy and drug screening

Tumor cell line expression profiles and mutation data were obtained from the Cancer Cell Line Encyclopedia (CCLE) website (https://portals.Broadinstitute.org/ccle/) (22). Data regarding the drug sensitivity of tumor cell lines in the CCLE database were retrieved from the Cancer Therapeutics Response Portal (CTRP, https://portals.broadinstitute.org/ctrp) and the Profiling Relative Inhibition Simultaneously in Mixtures website (PRISM, https://depmap.org/portal/prism/). The CTRP database contains data regarding the sensitivity of more than 835 tumor cell lines to 481 compounds, whereas the PRISM website provides access to data regarding the sensitivity of more than 482 tumor cell lines to 1,448 compounds. Both datasets provide the AUC as a measure of drug sensitivity, with lower AUC values indicating increased sensitivity to the drug treatment. The k-nearest neighbor model was used to input missing AUC values for the samples. As both datasets contain data regarding tumor cell lines from the CCLE project, CCLE data were used for the subsequent CTRP and PRISM analyses.

### 2.10 Statistical analysis

The TMB, MSI, neoantigen counts, overall immune scores of risk groups, expression levels of immune-related genes, and immune infiltration scores were compared between the mutant KRAS group and the WT KRAS group using the *t*-test. *P* values < 0.05 were considered to indicate statistically significant differences. All statistical tests in this study were two-sided tests. Moreover, all statistical tests and visualization were performed using R packages (version 3.6.1). The statistical parameters for box plot visualization were calculated using the R package ‘ggpurbr.’

## 3 Results

### 3.1 Tumor mutation profile and CNV analyses of different KRAS mutation groups

**Figure 1:**
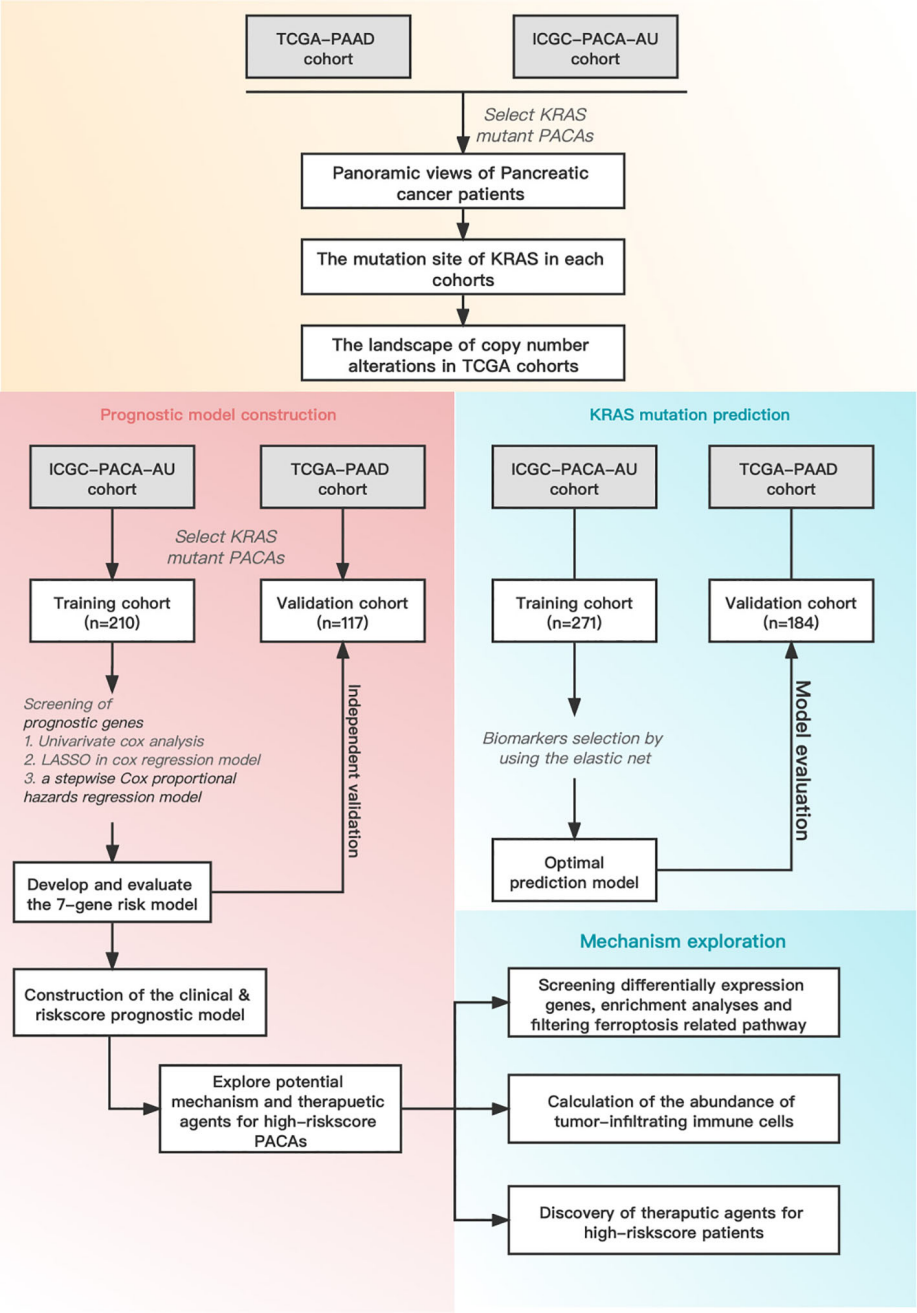
Schematic diagram of the overall study design and workflow.

**Figure 2:**
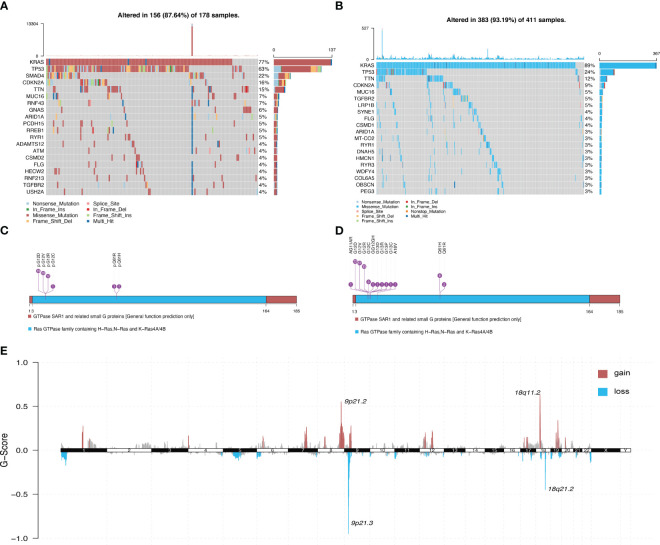
Panoramic view of mutations in the KRAS mutant group and KRAS wild-type group. **(A)** Distribution of mutations across different groups of patients with PDAC in the TCGA dataset. **(B)** Distribution of mutations in patients in the ICGC dataset. **(C)** Mutation sites on KRAS genes in PDAC samples in the TCGA dataset. **(D)** Mutation sites on KRAS genes in PDAC samples in the ICGC dataset. **(E)** Genome-wide distribution of chromosomal amplifications and deletions in the TCGA cohort of KRAS mutations.

**Figure 1** illustrates the overall workflow of this study. The clinical information contained in the datasets used in this study is summarized in **Supplementary Table 1**. We first analyzed the somatic mutations of PDAC samples in TCGA and ICGC datasets, then plotted waterfall charts to show differences in the mutation profiles of patients between different groups. The *KRAS* gene exhibited the highest mutation abundance, which far exceeded the other common genes in both datasets (**Figures 2A, B**). According to lollipop charts, the major mutation sites on the *KRAS* gene included G12D, G12V, and G12R (**Figures 2C, D**). CNV detection in the KRAS-mutant group using the GISTIC algorithm showed that deletions of 9q21.2 and 18p21.2, as well as amplifications of 18p11.2 and 18q11.2, were among the most significant chromosome arm-level CNVs in the KRAS-mutant group (**Figure 2E**).

### 3.2 Model for predicting KRAS mutations based on mRNA expression profiles

**Figure 3:**
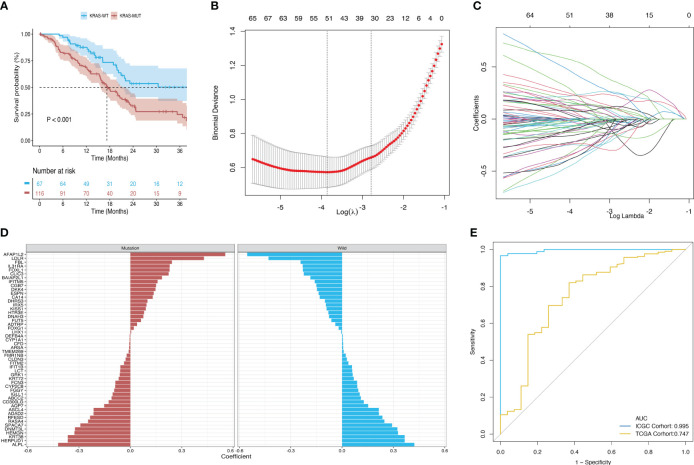
Construction and validation of a diagnostic model based on the expression signature of 50 genes. **(A)** Survival differences between the mutant and wild-type KRAS groups in the TCGA dataset. **(B, C)** Binomial deviance as a function of the regularization parameter (lambda) for leave-one-out cross-validation of the training dataset. Each dot in the graph corresponds to a classifier, and error bars represent the standard deviations. Coefficients of 50 genes were selected at the minimum value of lambda. **(D)** Coefficient for each of the 50 selected genes in each class, where a positive coefficient indicates that upregulated expression of the gene increases the probability of a sample belonging to this class. **(E)** ROC curves of classifiers based on the above genes and their external validation using an independent cohort.

Survival analysis of the TCGA-PAAD cohort revealed that patients in the KRAS-mutant group had poor overall prognosis (**Figure 3A**). Hence, we established a model for predicting mutations based on mRNA expression profiles. **Figure 3** shows that a binary classifier was identified for all samples in the training dataset using the minimum value of the regularization parameter given by ElasticNet (**Figure 3B**). This classifier was established based on the expression signatures of 50 genes (**Figure 3C**). Moreover, genes with non-zero coefficients were mutually exclusive for each class (**Figure 3D**). AUC values were calculated to evaluate the predictive performance for the training (ICGC) and validation (TCGA) datasets, which were 0.995 and 0.747, respectively (**Figure 3E**).

### 3.3 Construction of a prognostic model based on patients with KRAS mutations

**Figure 4:**
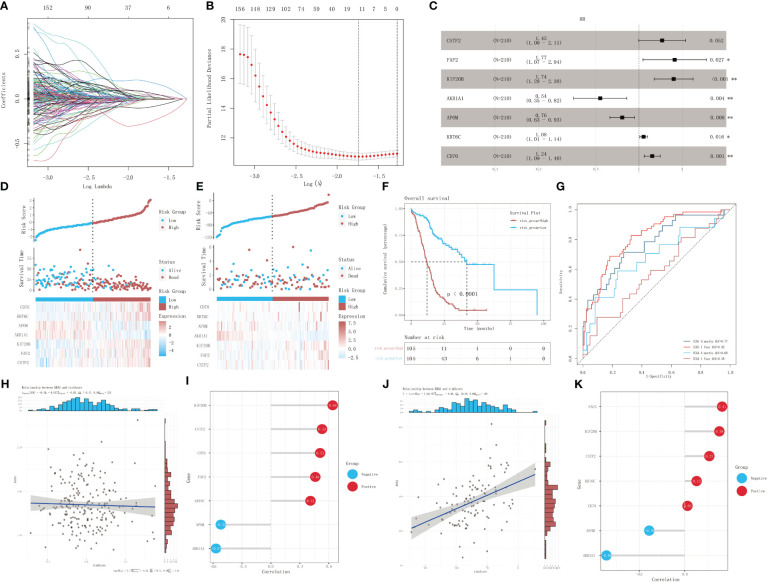
Identification of prognostic genes in patients with KRAS mutations. **(A)** A 100-fold cross-validation of parameter selection in the LASSO model. **(B)** LASSO coefficient profiles for the screening of prognostic genes. **(C)** Further screening of prognostic genes using a stepwise Cox proportional-hazards regression model. **(D)** Risk score distribution, survival, and gene expression profile of the ICGC cohort. **(E)** Risk score distribution, survival, and gene expression profile of the TCGA cohort. **(F)** Kaplan–Meier survival curves showing the survival difference between different risk-score groups in the training dataset. **(G)** ROC analysis of the prediction of 6-month and 1-year prognosis using the training dataset and validation dataset, respectively. **(H, I)** Correlation analysis of risk score and mRNA expression level of KRAS in the training set, and lollipop chart depicting the correlation between the risk score and the expression of genes selected for model construction. **(J, K)** Correlation analysis of risk score and mRNA expression of KRAS in the validation dataset, and lollipop chart depicting the correlation between the risk score and the expression of genes selected for model construction. *p<0.05; **p<0.01.

The univariate Cox proportional-hazards regression model based on the ICGC cohort of patients with KRAS mutations uncovered a total of 1,536 genes associated with OS (*p* < 0.05) (**Figure 4A**). We also screened for prognostic genes using a Lasso-Cox regression model according to the one-standard-error rule (**Figure 4B**). The model was optimized to include only the most predictive genes using a stepwise Cox proportional-hazards regression model. Finally, seven genes were included in the model: *CSTF2*, *FAF2*, *KIF20B*, *AKR1A1*, *APOM*, *KRT6C*, and *CD70* (**Figure 4C**). Patients in the ICGC training cohort (**Figure 4D**) and TCGA validation cohort (**Figure 4E**) were divided into a high-risk group and a low-risk group according to their optimized risk scores. Kaplan–Meier survival analysis showed that the high-risk group had a significantly lower survival rate than the low-risk group in the training cohort (**Figure 4F**, *p* < 0.0001). We then calculated the time-dependent AUC values for the two cohorts to evaluate the predictive performance of the model. The training cohort had AUC values of 0.77 and 0.82 for 6-month and 1-year survival predictions, respectively, whereas the validation cohort had AUC values of 0.68 and 0.58 for 6-month and 1-year survival prediction, respectively (**Figure 4G**). The correlation between the risk score and mRNA expression level of KRAS was validated using the training dataset and validation dataset, respectively. No significant correlation was detected between the risk score and KRAS expression in the training dataset (*p* = 0.637, R = -0.03) (**Figure 4H**); however, there was a strong positive correlation between the risk score and KRAS expression in the validation dataset (*p* < 0.001, R = 0.46) (**Figure 4I**). Correlation analysis between the risk score and the expression profiles of genes selected for model construction revealed the strongest positive correlation with KIF20B in the training dataset (**Figure 4J**) and FAF2 in the validation dataset (**Figure 4K**). The risk score exhibited the strongest negative correlation with AKR1A1 in both datasets.

### 3.4 Correlation between risk score and clinical parameters

**Figure 5:**
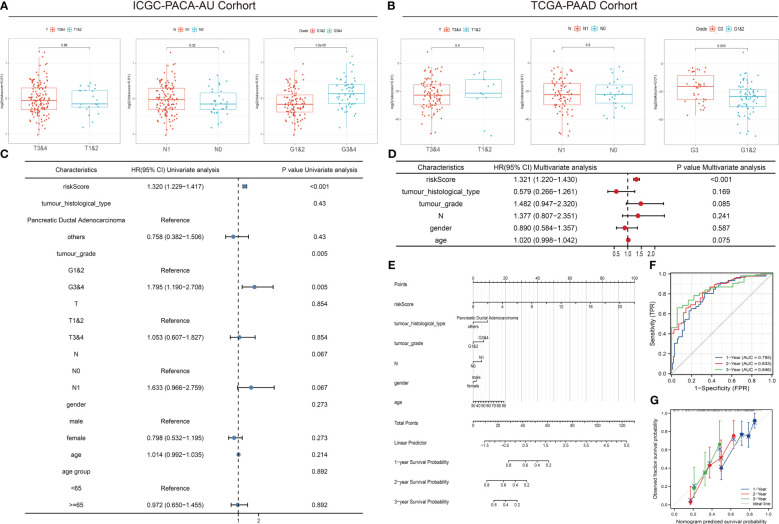
Correlation between risk scores, clinical characteristics, and independent prognostic factors. **(A, B)** Differences in clinical characteristics between risk groups in different cohorts. **(C, D)** Univariate and multivariate regression analyses of characteristics and other clinical factors associated with risk stratification. **(E)** Nomogram constructed based on clinical characteristics and risk scores for predicting the 1-year, 1-year, and 3-year survivals of PDAC patients with KRAS mutations. **(F)** ROC analysis of the prediction of 1-year, 2-year, and 3-year prognosis using the ICGC cohort. **(G)** Calibration curves showing the consistency between predicted and observed 1-year, 2-year, and 3-year survival rates. Gray solid line represents the perfect prediction made using the ideal model; other colored solid lines represent the actual performance of the nomogram, where a closer fit to the gray line represents a higher predictive accuracy.

The comparison of tumor stage, lymph node metastasis, and pathological grade between risk groups in the two datasets revealed that the high-risk group had a relatively lower pathological grade, with a statistically significant difference in the two cohorts (*p* < 0.001, *p* = 0.003) (**Table 1**; **Figures 5A, B**). Both univariate and multivariate regression analyses showed that the risk score had an excellent predictive ability for the OS and prognosis of patients with PDAC plus KRAS mutations in the ICGC cohort. In addition, the risk stratification based on age, gender, histological type, pathological grade, T stage, N stage, etc. (**Figures 5C, D**) did not identify any independent prognostic factor among these clinical factors. For quantitative prognostic prediction, the training cohort was used to establish the best multivariate model, which considers not only the risk score but also the histological type, pathological grade, N stage, gender, and age. The model is presented in the form of a nomogram in **Figure 5E**. The time-dependent AUC values of the model, calculated to assess its predictive performance, were 0.795, 0.833, and 0.846 for 1-year, 2-year, and 3-year survivals, respectively (**Figure 5F**). The calibration curves showed greater consistency between the prediction and ideal models for predicting the 1-year, 2-year, and 3-year prognoses using Linear Algebra and Machine Learning (LAML) (**Figure 5G**).

**Table 1:** Correlation between risk score and clinical parameters.

Characteristics	Total (N)	Univariate analysis		Multivariate analysis	
		Hazard ratio (95% CI)	P value	Hazard ratio (95% CI)	P value
Risk score	162	1.320 (1.229–1.417)	**<0.001**	1.321 (1.220–1.430)	**<0.001**
Tumor histological type	162				
PDAC	144	Reference			
other	18	0.758 (0.382–1.506)	0.430	0.579 (0.266–1.261)	0.169
Tumor grade	162				
G1&2	107	Reference			
G3&4	55	1.795 (1.190–2.708)	**0.005**	1.482 (0.947–2.320)	0.085
T	162				
T1&2	23	Reference			
T3&4	139	1.053 (0.607–1.827)	0.854		
N	162				
N0	37	Reference			
N1	125	1.633 (0.966–2.759)	0.067	1.377 (0.807–2.351)	0.241
Gender	162				
male	90	Reference			
female	72	0.798 (0.532–1.195)	0.273	0.890 (0.584–1.357)	0.587
Age	162	1.014 (0.992–1.035)	0.214	1.020 (0.998–1.042)	0.075
Age group	162				
<65	65	Reference			
≥65	97	0.972 (0.650–1.455)	0.892		

G1: well differentiated, low grade, mitotic rate<2, Ki-67 index<3%;

G2: well differentiated, intermediate grade, mitotic rate 2-20, Ki-67 index 3%-20%;

G3: well differentiated, high grade, mitotic rate>20, Ki-67 index>20%;

G4: poorly differentiated, high grade, mitotic rate>20, Ki-67 index>20%.

T1: Tumor ≤2 cm in greatest dimension.

T2: Tumor >2 cm and ≤4 cm in greatest dimension.

T3: Tumor >4 cm in greatest dimension.

T4: Tumor involves celiac axis, superior mesenteric artery, and/or common hepatic artery, regardless of size.

N0: No regional lymph node metastases.

N1: Metastasis in regional lymph nodes.

Bold values indicate statistical significance.

### 3.5 Correlation between the model and mutation-associated parameters

**Figure 6:**
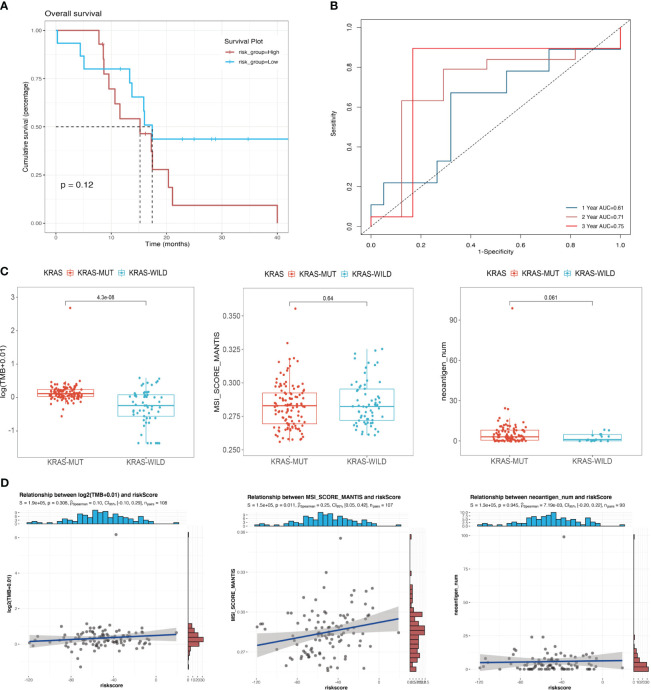
Correlation between mutational signatures and risk score. **(A)** Survival analysis of different risk groups in the TCGA cohort of KRAS wild-type patients; **(B)** ROC analysis of the prediction of 1-year, 2-year, and 3-year prognosis for the TCGA cohort of patients with wild-type KRAS. **(C)** Differences in TMB, MSI, and neoantigen counts between the KRAS wild-type group and the KRAS mutant group in the TCGA cohort. **(D)** Analysis of the correlation of risk score with TMB, MSI, and neoantigen counts in the TCGA cohort of patients with KRAS mutations.

We confirmed the effectiveness of the risk score for predicting the prognosis of patients with pancreatic cancer in the cohort of KRAS mutations. We also validated the predictive ability of the risk score using the TCGA cohort of WT KRAS, thereby demonstrating the extensiveness of the prediction model. The survival analysis (**Figure 6A**) revealed intersections between the two curves in the initial stage, whereas risk stratification provided a better prognostic prediction in the later stage. However, there was no statistically significant difference, which could be attributed to the limited sample size (*p* = 0.12). **Figure 6B** shows that KRAS WT patients in the TCGA cohort had similar results to patients with KRAS mutants with 1-year, 2-year, and 3-year AUC values of 0.61, 0.71, and 0.75, respectively. **Figure 6C** indicates a statistically significant difference in the TMB between the KRAS-mutant group and the KRAS WT group in the TCGA cohort (*p* < 0.001). However, unlike the TMB, the MSI score showed a significant positive correlation with the risk score in the cohort of KRAS mutations (*p* = 0.011, R = 0.25) (**Figure 6D**), indicating that the underlying mechanisms affecting prognosis, which were used to perform the risk stratification, may differ from the mechanisms by which KRAS mutations lead to poor prognosis.

### 3.6 DEGs and enriched pathways between different risk groups in the cohort of patients with KRAS mutations

**Figure 7:**
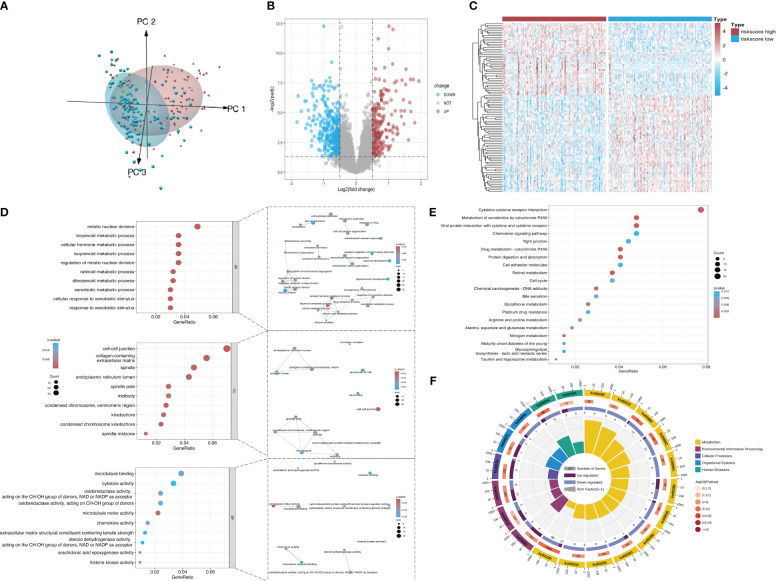
Screening of DEGs from the datasets. **(A)** Principal component analysis of mRNA expression matrices in the ICGC datasets of KRAS mutations. **(B)** Volcano plot of all mRNAs in the dataset. Red and dark blue dots represent significantly upregulated and downregulated genes in the high-risk group, respectively. **(C)** Heatmap showing the distribution of DEGs across the two groups. Red and dark blue boxes represent genes with high and low expression, respectively. **(D)** Significantly enriched GO terms and functional networks of GO terms. **(E)** Significantly enriched signaling pathways. **(F)** Donut chart of KEGG pathway enrichment. The first ring shows the top 20 most significantly enriched KEGG pathways with a coordinate ruler representing the number of genes outside the ring. Different colors represent different KEGG categories. The second ring shows the number of background genes in the category and the Q value or P value. The length of each bar indicates the number of genes, where a redder bar represents a smaller Q value or P value. The third ring is a bar chart showing the proportion of upregulated (dark purple) and downregulated (light purple) genes. The fourth ring shows the Rich Factor value of each category (number of foreground genes in the category divided by the number of background genes).

According to principal component analysis (**Figure 7A**), the two risk groups can still be readily distinguished from each other, despite some overlaps. DEGs between the high-risk group and the low-risk group were extracted from the ICGC-PACA-AU gene expression matrix using R software (refer to volcano plot and heatmap in **Figures 7B, C**) and subjected to pathway enrichment analysis and GO enrichment analysis. GO enrichment analysis divides GO terms into three ontologies: biological processes (BP), cellular components (CC), and molecular functions (MF). According to the results of GO enrichment analysis and the GO term networks, “metabolic process” was an important BP term among the DEGs, wherein the xenobiotic metabolic process is closely associated with ferroptosis (**Table 2**; **Supplementary Table 2**; **Figure 7D**). However, there was no significant enrichment of CC or MF terms.

**Table 2:** Top three most significantly enriched BP, CC, and MF terms in the GO enrichment analysis of DEGs between different risk groups.

Pathway ID	Category	Description	Count	p.adjust
GO:0006721	BP	terpenoid metabolic process	19	8.24E-06
GO:0034754	BP	cellular hormone metabolic process	19	1.29E-05
GO:0001523	BP	retinoid metabolic process	17	1.29E-05
GO:0005911	CC	cell-cell junction	39	3.62E-06
GO:0062023	CC	collagen-containing extracellular matrix	31	0.000272664
GO:0000779	CC	condensed chromosome, centromeric region	15	0.000272664
GO:0003777	MF	microtubule motor activity	12	0.001569573
GO:0008017	MF	microtubule binding	21	0.010928871
GO:0016616	MF	oxidoreductase activity, acting on the CH-OH group of donors, NAD or NADP as acceptor	13	0.010928871

The pathway enrichment analysis (**Table 3**) showed that the DEGs were mainly enriched in cytokine-cytokine receptor interaction, metabolism of xenobiotics by cytochrome P450, and viral protein interaction with cytokine and the cytokine receptor (**Figure 7E**), wherein metabolism of xenobiotics by cytochrome P450 is often associated with ferroptosis. The donut chart of pathway enrichment analysis (**Figure 7F**) shows that most of the enriched pathways are metabolic pathways predominantly enriched by downregulated genes, suggesting numerous downregulated metabolic pathways in the high-risk group. However, further analyses are still required to determine whether the high-risk group exhibited reduced ferroptosis that led to a poor prognosis.

**Table 3:** Top 10 most significant pathways enriched by DEGs between different risk groups.

ID	Description	Count	p.adjust
hsa00980	Metabolism of xenobiotics by cytochrome P450	13	0.000436743
hsa00982	Drug metabolism - cytochrome P450	11	0.002433605
hsa04061	Viral protein interaction with cytokine and cytokine receptor	13	0.002433605
hsa00830	Retinol metabolism	10	0.005496136
hsa04974	Protein digestion and absorption	11	0.035216816
hsa04060	Cytokine-cytokine receptor interaction	21	0.040369276
hsa05204	Chemical carcinogenesis - DNA adducts	8	0.073437966
hsa00910	Nitrogen metabolism	4	0.073437966
hsa00480	Glutathione metabolism	7	0.095520623
hsa00430	Taurine and hypotaurine metabolism	3	0.142513801

### 3.7 GSEA of all genes

**Figure 8:**
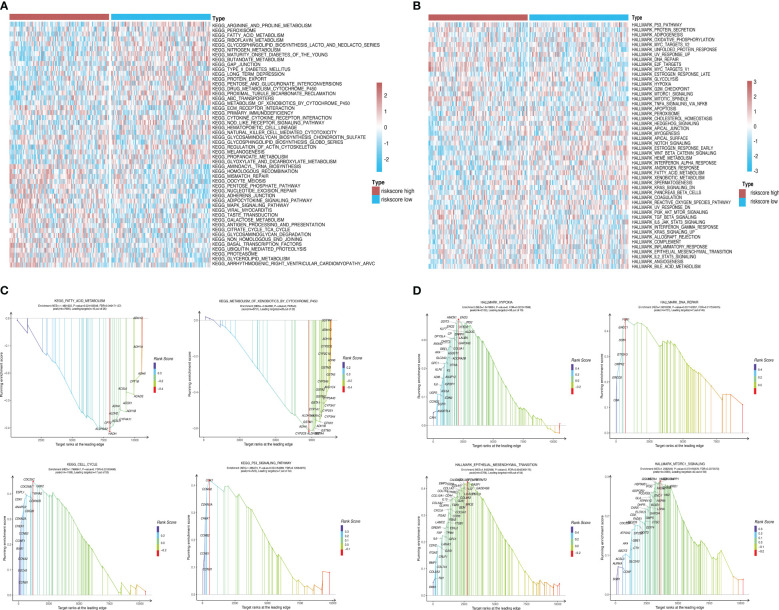
Pathway enrichment analysis between risk groups. **(A, B)** Heatmaps showing significant differential pathways between risk groups in c2.cp.kegg.v7.0.symbols.gmt and h.all.v7.2.symbols.gmt pathway sets, respectively. **(C, D)** GSEA of ferroptosis-related pathways reported in the literature and genes involved in these pathways.

Using GSEA, we further explored specific signaling pathways among the different risk groups in the ICGC dataset. As shown in **Tables 4**, **5**; **Supplementary Tables 3, 4**; **Figure 8A**, the high-risk group had a higher number of downregulated metabolic pathways than the low-risk group. Among these, fatty acid metabolism is closely associated with ferroptosis. Moreover, the high-risk group showed significantly upregulated expression of genes associated with the classical pathways of tumorigenesis (i.e., MAPK pathway and DNA damage-related pathways such as mismatch repair, homologous recombination, etc.). **Figure 8B** also shows similar results, wherein the P53 pathway, DNA repair pathways, etc., were upregulated in the high-risk group. Here, all ferroptosis-associated pathways reported in previous literature were retrieved for analysis. As shown in **Figures 8C, D**, fatty acid metabolism and xenobiotic metabolism were downregulated, whereas other oncogenic pathways (e.g., the cell cycle pathway, P53 pathway, and epithelial–mesenchymal transition (EMT) pathway) were upregulated in the high-risk group. Thus, the DEGs in these pathways were subsequently validated.

**Table 4:** Top 10 most significantly enriched hallmark gene sets in the GSEA between different risk groups.

Description	setSize	enrichmentScore	NES	FDR q-val
HALLMARK_HYPOXIA	106	0.37231782	1.6418664	0.022341566
HALLMARK_TNFA_SIGNALING_VIA_NFKB	113	0.39980704	1.7355567	0.011287187
HALLMARK_EPITHELIAL_MESENCHYMAL_TRANSITION	103	0.42478487	1.8432508	0.004236918
HALLMARK_MITOTIC_SPINDLE	112	0.5257801	2.3399587	0
HALLMARK_E2F_TARGETS	85	0.57736474	2.440232	0
HALLMARK_G2M_CHECKPOINT	97	0.58410454	2.5336802	0
HALLMARK_MYC_TARGETS_V1	95	0.34807268	1.4886647	0.06445707
HALLMARK_INTERFERON_GAMMA_RESPONSE	98	0.34494698	1.4875005	0.05810461
HALLMARK_DNA_REPAIR	52	0.445993	1.6810238	0.017534915
HALLMARK_PANCREAS_BETA_CELLS	18	-0.57997334	-1.6499071	0.06562658

**Table 5:** Top 10 most significantly enriched KEGG pathways in the GSEA between different risk groups.

Description	setSize	enrichmentScore	NES	FDR q-val
KEGG_CELL_CYCLE	72	0.44863206	1.7996947	0.08955626
KEGG_UBIQUITIN_MEDIATED_PROTEOLYSIS	57	0.43718052	1.7014115	0.12950997
KEGG_METABOLISM_OF_XENOBIOTICS_BY_CYTOCHROME_P450	48	-0.63126934	-2.244582	0
KEGG_DRUG_METABOLISM_CYTOCHROME_P450	48	-0.62915623	-2.2343724	0
KEGG_GLUTATHIONE_METABOLISM	37	-0.6026078	-2.032244	0.002371647
KEGG_VASOPRESSIN_REGULATED_WATER_REABSORPTION	32	-0.55526716	-1.8053486	0.029310916
KEGG_RETINOL_METABOLISM	36	-0.53703487	-1.8158599	0.03257731
KEGG_ECM_RECEPTOR_INTERACTION	64	0.41171035	1.6640197	0.11847458
KEGG_BUTANOATE_METABOLISM	29	-0.52998686	-1.7029611	0.06348657
KEGG_NITROGEN_METABOLISM	22	-0.60035735	-1.7704971	0.03784737

### 3.8 Correlation between risk score and immune microenvironment

**Figure 9:**
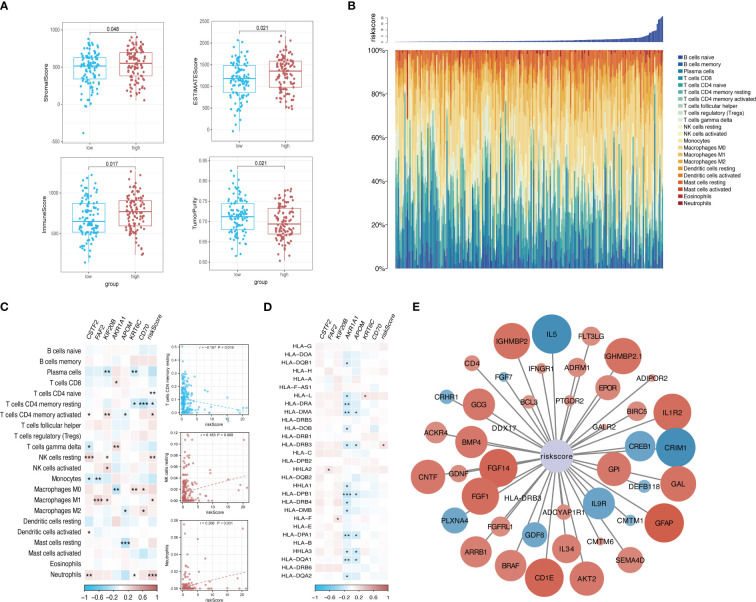
Risk-based immune microenvironment assessment. **(A)** Differences in immune microenvironment scores between risk groups. **(B)** Sorted histogram of risk scores showing the distribution of 22 types of infiltrating immune cells. **(C, D)**. Heatmap depicting the correlation of candidate core genes with the infiltration of 22 types of immune cells and the expression of HLA family genes. Red and blue boxes represent positive and negative correlations, respectively. Color intensity indicates the strength of the correlation; *p < 0.05; **p < 0.01; ***p < 0.001. **(E)** Correlation network of the risk score and 44 immune-related genes. Larger nodes are indicative of smaller P values. Red and blue nodes indicate positive and negative correlations, respectively. Color intensity indicates the strength of the correlation.

The tumor purity, stromal score, and immune score were determined using the ESTIMATE algorithm to investigate the differences between the low-risk group and the high-risk group. We observed significant differences in all four scores between the two risk groups (**Figure 9A**). We then sorted the samples in the ICGC cohort of patients with KRAS mutations by risk score and constructed a histogram to display the distribution of infiltrating immune cells in each sample. The results showed that the infiltration of some immune cells (e.g., neutrophils) increased as the risk score increased (**Figure 9B**). In addition, the correlations between the risk score and corresponding genes with the infiltration of 22 types of immune cells and the expression of HLA family genes were illustrated using correlation heatmaps. Neutrophil infiltration was significantly positively correlated with the risk score and was significantly negatively correlated with the infiltration of resting memory CD4 T cells and resting natural killer (NK) cells (**Figures 9C, D**). A total of 44 immune-related genes that were significantly correlated with the risk score were selected via correlation analysis and displayed as a correlation network (**Figure 9E**).

### 3.9 mRNA expression levels related to prognosis model in pancreatic cancer cell lines with different mutation sites

**Figure 10:**
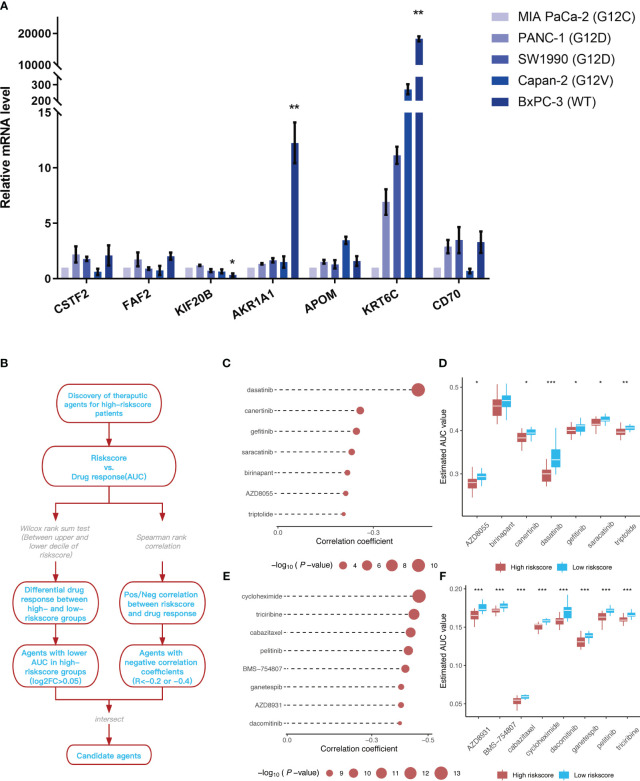
Identification of 7 model genes in pancreatic cancer cell lines and drug candidates exhibiting higher sensitivity in patients with high-risk scores. **(A)** Expression of CSTF2, FAF2, KIF20B, AKR1A1, APOM, KRT6C, and CD70 in human pancreatic cell lines and pancreatic cancer cell lines. **p < 0.05 BxPC-3 vs other pancreatic cancer cell lines; **p < 0.01 BxPC-3 vs other pancreatic cancer cell lines (*n*=3). **(B)** Overview of the workflow for identifying drugs with higher sensitivity in patients with high-risk scores. **(C, D)** Spearman’s correlation analysis and differential drug response analysis of seven compounds from the CTRP database. **(E, F)** Spearman’s correlation analysis and differential drug response analysis of eight compounds from the PRISM database. Note: Lower values on the y-axis of the boxplot are indicative of greater drug sensitivities. *p < 0.05; **p < 0.01; ***p < 0.001.

The mRNA expression levels of *CSTF2*, *FAF2*, *KIF20B*, *AKR1A1*, *APOM*, *KRT6C*, and *CD70*, which were included in the prognostic model, were detected in different types of pancreatic cancer cell lines (**Figure 10A**). Similar to the training dataset and validation dataset, *KIF20B* was expressed at a relatively low level in WT pancreatic cancer cells BxPC-3 (*p* < 0.05), while *AKR1A1* was highly expressed in WT pancreatic cancer cells (type BxPC-3), compared with mutant types, such as MIA Paca-2(KRAS G12C mut), PANC-1(KRAS G12D mut), SW1990(KRAS G12D mut) and Capan-2(KRAS G12V mut)(*p* < 0.01). Interestingly, *KRT6C* was extremely highly expressed in WT pancreatic cancer cells BxPC-3 (*p* < 0.01). As for *FAF2*, there was no statistical difference in the expression levels across different cell lines, reflecting the differences from the training dataset and validation dataset.

### 3.10 Screening of potential drugs related to the risk score

The CTRP and PRISM datasets, which contain gene expression profiles and drug sensitivity profiles for hundreds of tumor cell lines, can be used to establish a model that predicts the drug response. There are 160 overlapping compounds between the two datasets, with 1,770 compounds remaining after removing duplicates. Compounds containing nucleosides or nucleoside (acid) analogs, as well as cell lines derived from hematopoietic and lymphoid tissues, were excluded from the datasets. Finally, we analyzed the sensitivity data of 354 tumor cell lines to 354 compounds in the CTRP dataset and the sensitivity data of 439 tumor cell lines to 1,291 compounds in the PRISM dataset. Two different approaches were employed to identify drug candidates that demonstrated higher sensitivity in cell lines with high-risk scores, representing a phenotype closely aligned with high-risk patients (**Figure 10B**). First, the differential drug response analysis of patients with a high-risk score (upper decile) and a low palliative performance scale score (lower decile) was performed to identify potential compounds (LOG2FC > 0.05) in the high-risk group. Subsequently, the correlation between AUC values and risk score was analyzed to select compounds with negative Spearman’s correlation coefficients (R < -0.4 for the PRISM dataset and R < -0.2 for the CTRP dataset). These analyses gave rise to seven compounds from the CTRP database (triptolide, AZD8055, birinapant, saracatinib, gefitinib, canertinib, and dasatinib) and eight compounds from the PRISM database (dacomitinib, AZD8931, ganetespib, BMS-754807, pelitinib, cabazitaxel, riciribine, and cycloheximide) (**Figures 10C-F**).

## 4 Discussion

KRAS mutations in patients with PDAC affect the selection of drugs and patient prognosis (23–25). Therefore, research into KRAS mutations in patients with PDAC will improve our understanding of their roles in the onset and development of PDAC. Previous studies have suggested that the development, metastasis, and therapeutic resistance of PDAC are predominantly controlled by KRAS. Various approaches to tackling RAS mutations have been employed, including the direct inhibition of RAS, prevention of its membrane localization, and inhibition of its upstream or downstream signaling molecules (25–27). However, only second-generation EGFR inhibitors targeting the upstream EGFR of RAS and therapeutic methods targeting the downstream RAF-MEK-ERK pathway have exhibited any therapeutic effects (28, 29). Previous studies, as well as the analysis in this study, have uncovered ubiquitous KRAS^G12D^, KRAS^G12V^, and KRAS^G12R^ point mutations in patients with PDAC (30). Current therapeutic approaches for cancer, e.g., monochemotherapy targeting KRAS mutations and a combination of chemotherapy with radiotherapy/immunotherapy, have exhibited limited efficacy in patients with PDAC (31). One important reason is the development of drug resistance, especially apoptosis resistance. Ferroptosis is one of many non-apoptotic forms of cell death mediated by lipid peroxidation. Therefore, non-apoptotic types of cell death, e.g., ferroptosis, may offer new therapeutic strategies against PDAC or apoptosis resistance (32). Considering the fact that KRAS mutations profoundly affect patient prognosis, and that any single factor may not be sufficient for classifying patients with PDAC, we explored the possibility of predicting PDAC prognosis based on KRAS mutations, ferroptosis-associated pathways, and the immune microenvironment.

In this study, we found that patients with KRAS^G12D^, KRAS^G12V^, and KRAS^G12R^ mutations had poor prognosis, which is consistent with the results of previous studies (9, 33). Hence, it is necessary to determine the status of KRAS mutations in patients before treatment. However, the diagnosis of KRAS mutations via surgical resection and biopsy has a limited detection rate (34, 35). Moreover, tumor heterogeneity may affect the diagnostic accuracy. As KRAS mutations can also be predicted from the expression of other genes, we constructed and validated a model for predicting KRAS mutations based on mRNA levels, which exhibited better predictive performance than diagnosis via surgical resection alone. Previous studies have also demonstrated excellent predictive performance of data analysis and machine learning for KRAS mutations in colon and lung cancers (10). To the best of our knowledge, the present study is the first to construct a model for predicting KRAS mutations in PDAC. We reported AUC values of 0.995 and 0.747 for the training and validation datasets, respectively. These values indicate strong discriminative ability of our model. Comparisons of these AUC values with those reported in other reference studies (36, 37) provide further insights into the exceptional predictive performance of our model. However, it is important to acknowledge that our model’s use of data was limited to Black and White American populations, based on data availability. Our analysis primarily relied on a dataset containing information predominantly from these two racial/ethnic groups, shaping the foundation of our findings and conclusions. It is crucial to recognize that addressing racial and ethnic disparities in cancer research is a complex issue, and our study’s focus on these populations should not overshadow the significance of studying other racial and ethnic groups in understanding PDAC prognosis. Further research including diverse populations will be necessary to comprehensively address this important aspect. Regarding our predictive model, it specifically focuses on estimating the time-dependent AUC values for 1-year, 2-year, and 3-year survival rates in PDAC patients. These specific time intervals are commonly employed in assessing prognosis and treatment response in PDAC, given the low 5-year survival rate associated with the disease. By selecting these particular time points, our aim is to provide a comprehensive understanding of the model’s ability to predict short-term and medium-term outcomes. Furthermore, these time intervals align with standard clinical evaluations and enable meaningful comparisons with other studies conducted in the field.

To further determine prognostic genes in patients with KRAS mutations, we assessed the predictive performance of seven prognostic genes screened using a Lasso-Cox regression model (*CSTF2*, *FAF2*, *KIF20B*, *AKR1A1*, *APOM*, *KRT6C*, and *CD70*), as well as their correlation with the mRNA expression level of *KRAS*. The selection of the alpha parameter in elastic net regularization is crucial in building the prediction model. Alpha regulates the trade-off between L1 (Lasso) and L2 (Ridge) regularization, with 1 denoting Lasso regression, 0 representing Ridge regression, and any value between 0 and 1 indicating a combination of the two. In this study, an alpha value of 0.9 was chosen for various reasons. First, it promotes feature selection by encouraging sparsity in the model, selecting a subset of highly relevant features while penalizing less informative ones. This is particularly useful in the intricate domain of genomic data for identifying crucial genes associated with KRAS mutations. Additionally, higher alpha values (e.g., 0.9) diminish the risk of overfitting, which is especially critical when dealing with biological data having a higher number of features (genes) than samples. It helps prevent the model from fitting noise in the data. Stepwise Cox regression is used to select Kras genes closely related to survival outcomes. It enhances the model through feature selection, model parsimony, improved predictive capacity, and identifying the optimal gene subset. The results revealed that *KIF20B* and *FAF2* displayed the strongest positive correlation in the training dataset and validation dataset, respectively. However, *AKR1A1* had the strongest negative correlation with the risk score in both datasets. A bioinformatics analysis performed by Yang et al. showed that KIF20B may be a prognostic marker of PDAC (38). This finding was then experimentally confirmed by Chen et al. (39). A bioinformatics analysis conducted by Bai et al. on DEGs associated with lipid metabolism revealed that *FAF2* was highly expressed in pancreatic cancer tissues and cells, and that abnormalities in lipid metabolism are an important ferroptosis-associated pathway (40), laying the foundations for this study. CD70, which is a cytokine belonging to the tumor necrosis factor ligand family, is also a prognostic marker for pancreatic cancer (41) and a potential therapeutic target for pancreatic cancer (42). AKR1A1 is an aldehyde reductase that is associated with resistance to radiotherapy and chemotherapy in laryngeal cancer, breast cancer, etc.; however, there have been no similar studies for pancreatic cancer. Indeed, there are extremely few high-quality mechanistic studies on other signature genes (e.g., *CSTF2*, *APOM*, and *KRT6C*) that may serve as targets for subsequent studies on the molecular mechanisms of pancreatic cancer. Importantly, the correlation between the risk score model and previously identified KRAS mutations further confirmed the association between these variables. The excellent predictive performance of nomograms combining the seven signature genes and clinicopathological parameters may allow clinicians to better determine the prognosis of individual patients.

According to the GO enrichment and network analyses of DEGs in the KRAS-mutant group, “metabolic process” was an important BP term enriched by the DEGs, and xenobiotics play an important role in KRAS mutations. A xenobiotic is a chemical substance found within an organism but not usually produced or expected to be present within the organism (43). The production of xenobiotics in an organism can also be considered as a response to foreign substances (e.g., drugs). The pathway enrichment analysis also confirmed that xenobiotics may affect ferroptosis by altering the metabolism of the cytochrome P450 system. The GSEA uncovered multiple altered ferroptosis-associated metabolic pathways in the high-risk group, among which the downregulation of fatty acid metabolism and xenobiotic metabolism further demonstrated the roles of xenobiotics identified via GO and KEGG enrichment analyses. Other oncogenic pathways (e.g., -The drug response of PDAC cells is greatly affected by metabolic processes (8). Despite alleviating apoptosis, SLC2A1-mediated glucose uptake can promote the ferroptosis induced by System X_c_^-^ inhibitors (but not that induced by GPX4 inhibitors) in various human cell lines or primary cells of PDAC, because System X_c_^-^ inhibitors, rather than GPX inhibitors, selectively suppress the expression of PDK4 (44). The latter can block ferroptosis in PDAC cells (PANC1 and MIAPaCa2) by inhibiting pyruvate oxidation in mitochondria via the phosphorylation of pyruvate dehydrogenase (PDH) (45). Metabolic assays have demonstrated that pyruvate oxidation generates acetyl-CoA in PANC1 cells for subsequent fatty acid synthesis catalyzed by acetyl-CoA carboxylase alpha (ACACA) and fatty acid synthase (FASN) (46). Eventually, the increased level of fatty acids provides more substrates for ALOX5-mediated lipid peroxidation under oxidative stress (47). In contrast to the pyruvate oxidation- or glutaminolysis-mediated pro-ferroptotic properties of mitochondria, branched-chain amino acid transaminase 2 (BCAT2)-mediated glutamate conversion and the subsequent GSH synthesis inhibit System X_c_^-^ inhibitor-induced ferroptosis (44). Furthermore, ferritin phagocytosis and subsequent activation of the AMPK pathway can suppress the expression of BCAT2 in the pancreatic cancer cell line AsPC-1, suggesting that AMPK plays a dual role in ferroptosis (44). The dual effects of fatty acid metabolism rely on the type of tumors and stimulation by drugs (e.g., xenobiotics). *KRAS* and *p53* are the most common mutated genes in PDAC (48). Kim et al. found that KRAS mainly mediates the phosphorylation of CREB1S133, and that activated CREB1 can enhance the binding affinity of mutant p53 for the FOXA1 promoter, thereby activating its transcriptional network and promoting the Wnt/β-catenin signaling pathway, which jointly drives the metastasis of PDAC (8). p53 is an important regulator of ferroptosis that exhibits broad and complex roles, not only in canonical GPX4-mediated ferroptosis but also in non-canonical GPX4-independent ferroptosis pathways (49). Future studies on the roles of KRAS and p53 in ferroptosis will provide new insights into the mechanism of ferroptosis as well as the treatment of PDAC by targeting KRAS, p53, and ferroptosis.

The proportions of immune and stromal components in the tumor microenvironment of each sample were estimated using the ESTIMATE algorithm and presented as the immune score, stromal score, and ESTIMATE score, which are positively correlated with the infiltration of immune cells, the presence of stromal cells, and the sum of the immune score and stromal score, respectively (i.e., high scores denote greater proportions of corresponding components in the tumor microenvironment). We found that the high-risk group had a higher immune score and stromal score, as well as poorer OS, suggesting that the risk score model in this study can be used to accurately stratify patients based on their immune microenvironment. Immune cell infiltration analysis showed that the level of infiltrating neutrophils increased with an increasing risk score and had a significant negative correlation with resting memory CD4 T cells and resting NK cells, indicating that neutrophil inhibition can hinder the progression of high-risk pancreatic cancer. Moreover, lorlatinib can prevent the growth of pancreatic cancer by suppressing tumor-associated neutrophils and improve prognosis with immune checkpoint blockade (50). The inhibition of dipeptidyl peptidase can alter the CXCR3 axis and enhance the infiltration of NK cells to improve the anti-PD1 effect in mouse models of PDAC (51). Our findings are consistent with those of the above studies, but still require further experimental confirmations in humans. The subsequent correlation analysis identified immune-related genes that are significantly correlated with the risk score, among which the positively correlated gene *FGF14* was significantly correlated with the prognosis of pancreatic cancer (52); other positively and negatively correlated genes still require further experimental validation because of a lack of high-quality research on their basic correlation with PDAC.

The development of targeted drugs to inhibit RAS-driven cancers has faced many challenges. Mutated KRAS is known as an “undruggable” target because of its high binding affinity for GTP, small catalytic sites, and smooth protein surface. However, previous experience in the development of KRAS^G12C^ inhibitors has provided lead compounds that aid the development of drugs targeting other KRAS mutations [e.g., KRAS^G12D^, KRAS^G12V^, and KRAS^G12R^ (10)]. The development and incorporation of KRAS^G12C^-specific inhibitors, like MRTX849, provide new opportunities for targeted therapy against previously considered ‘undruggable’ KRAS mutations. Future studies should continue to investigate the efficacy and safety profiles of MRTX849 and other emerging KRAS mutant inhibitors, further exploring their potential in combination therapies and understanding mechanisms of resistance (10). In the present study, after detecting the mRNA expression levels related to the prognosis model in pancreatic cancer cell lines with different mutation sites, we confirmed the consistency of this model in cell lines. Then, using a comparison of KRAS mutations between the high-risk group and the low-risk group, we identified a total of 15 highly effective drugs in the two chemical databases, among which dacomitinib, pelitinib, dacomitinib, and pelitinib are EGFR pathway inhibitors whose effects on pancreatic cancer cells have been previously reported (28). Some of these drugs have even entered phase I (53, 54) and phase II (53) clinical trials. For example, ganetespib has entered a phase II clinical trial (55); however, other drugs have only been investigated in fundamental studies on pancreatic cancer cells, and hence the supporting data from clinical trials are lacking (56). Nevertheless, the therapeutic potentials of AZD893 and riciribine against PDAC have not yet been reported and require further investigation. Recently, Jiang et al. (57) successfully converted an FDA-approved MEK inhibitor into a ferrous iron-activatable drug conjugate (FeADC), which showed potent MAPK blockade in tumor cells with KRAS mutations, while sparing normal tissues. This demonstrates that ferrous iron (Fe^2+^) accumulation is an exploitable feature for transforming drugs to target mutated KRAS, and that FeADC holds potential for improving the treatment of KRAS-driven solid tumors. Our study demonstrates the clinical applications of compounds from the CTRP and PRISM databases provide effective strategies and circumstantial evidence for the screening of drugs targeting KRAS mutations. These databases offer opportunities for drug repurposing, identification of combination therapies, personalized medicine approaches, and biomarker discovery (58). As for other genes whose expression in cell lines was inconsistent with the model, we hope that further verification can be carried out by other means, such as organoid models, in which the sensitivity of candidate drugs can be further verified.

In conclusion, we established, analyzed, and validated a model for predicting the prognosis of PDAC based on risk stratification according to KRAS mutations, as well as identified differential pathways and highly effective drugs, laying the foundation for subsequent development of drugs targeting PDAC with KRAS mutations. However, our study has certain limitations: (1) As all information and tissues were retrieved retrospectively from public databases, our external validation could not cover all changes in PDAC cases in all relevant regions; (2) Some of the observed differences were not statistically significant because of an insufficient number of patients with pancreatic cancer in the TCGA database; and (3) The KRAS mutation status in different regions of a tumor might be indistinguishable when considering the tumor as a monolithic entity because of intratumor heterogeneity between the inner and outer region of the tumor. A combination of single-cell RNA sequencing with spatial transcriptome analysis could be employed in subsequent studies to address the issues related to possible intratumor heterogeneity.

## Data availability statement

The original contributions presented in the study are included in the article/**Supplementary Material**. Further inquiries can be directed to the corresponding authors.

## Ethics statement

Ethical approval was not required for the studies on humans in accordance with the local legislation and institutional requirements because only commercially available established cell lines were used.

## Author contributions

FaY and SS designed the study. YH and FeY integrated and analyzed the data. NG and JG prepared the figures. FaY and YH wrote the manuscript. SS supervised the study. All authors contributed to the article and approved the submitted version.
