# Supplementary material for: Exploring KRAS-mutant pancreatic ductal adenocarcinoma: a model validation study
Source: Front Immunol. 2024 Jan 10;14:1203459. doi: 10.3389/fimmu.2023.1203459 (PMC10805828; doi:10.3389/fimmu.2023.1203459)
Supplement: Supplementary file 1 [file DataSheet_1.docx]

Supplementary Materials

# Supplementary Materials

## Cell culture

MIA-PaCa-2, PANC-1 and BxPC-3 cells were cultured in Dulbecco’s modiﬁed Eagle’s medium (DMEM) (Gibco, Gaithersburg, MD, United States); Capan-2 cells were cultured in RPMI-1640 medium (Gibco, Gaithersburg, MD, United States); and SW1990 cells were cultured in Leibovitz's L-15 medium (Gibco, Gaithersburg, MD, United States). All media contained 10% fetal bovine serum (FBS) (Gibco, Gaithersburg, MD, United States) and all cells were cultured in a humidiﬁed atmosphere consisting of 5% CO_2_ and 95% air at 37 ℃.

## Total RNA extraction

Total RNA from all cell lines was extracted using TRIzol reagent (Invitrogen, Carlsbad, CA, United States). Quantity and quality of RNA were determined spectrophotometrically at 260 nm and 280 nm. The integrity and contamination were conﬁrmed using denaturing agarose gel electrophoresis.

## Reverse transcription

Total RNA was reverse transcribed using a PrimeScript reagent kit with gRNA Eraser (Random primers) (TaKaRa, Dalian, China) according to the manufacturer’s instructions.

## CSTF2, FAF2, KIF20B, AKR1A1, APOM, KRT6C, and CD70 detection using qRT-PCR

qRT-PCR was performed using a Roche 480II system (Roche, Basel, Switzerland) utilizing SYBR Premix Ex Taq Ⅱ (Tli RNaseH Plus) (Takara, Dalian, China), following the manufacturer-provided instructions. Primers for GAPDH and other mRNA were synthesized by Sangon Biotech (Shanghai, China) as shown in Table S5. Expressions were normalized to endogenous controls and fold change was determined as 2^-ΔΔCT^ in mRNA expression after three independent experiments. All results are expressed as mean ± SD.

# Supplementary Tables

## Clinical characteristics of PDAC in the datasets used in this study

Table S1. Clinical characteristics of PDAC in the datasets used in this study

| Characteristic | TCGA KRAS mutations | ICGC KRAS mutations | *p* value |
| --- | --- | --- | --- |
| Number (n) | 117 | 210 |  |
| stage, n (%) |  |  |  |
| STAGE IA | 2 (1.7%) |  |  |
| STAGE IB | 6 (5.1%) |  |  |
| STAGE IIA | 22 (18.8%) |  |  |
| STAGE IIB | 80 (68.4%) |  |  |
| STAGE III | 3 (2.6%) |  |  |
| STAGE IV | 4 (3.4%) |  |  |
| Neoplasm histologic grade, n (%) |  |  | 0.174 |
| 1 | 11 (3.9%) | 8 (2.9%) |  |
| 2 | 66 (23.6%) | 100 (35.7%) |  |
| 3 | 38 (13.6%) | 53 (18.9%) |  |
| 4 | 0 (0%) | 4 (1.4%) |  |
| N, n (%) |  |  | 0.801 |
| N0 | 29 (10.4%) | 38 (13.6%) |  |
| N1 | 86 (30.8%) | 126 (45.2%) |  |
| T, n (%) |  |  | 0.708 |
| T1 | 2 (0.7%) | 1 (0.4%) |  |
| T2 | 12 (4.3%) | 22 (7.8%) |  |
| T3 | 101 (35.8%) | 140 (49.6%) |  |
| T4 | 2 (0.7%) | 2 (0.7%) |  |
| Race category, n (%) |  |  | 1.000 |
| Asian | 10 (8.9%) | 0 (0%) |  |
| Black or African American | 3 (2.7%) | 0 (0%) |  |
| White | 99 (88.4%) | 0 (0%) |  |
| Sex, n (%) |  |  | 0.432 |
| Female | 48 (14.7%) | 97 (29.7%) |  |
| Male | 69 (21.1%) | 113 (34.6%) |  |
| Age, median (IQR) | 66 (56, 74) | 67 (60, 75) | 0.265 |
